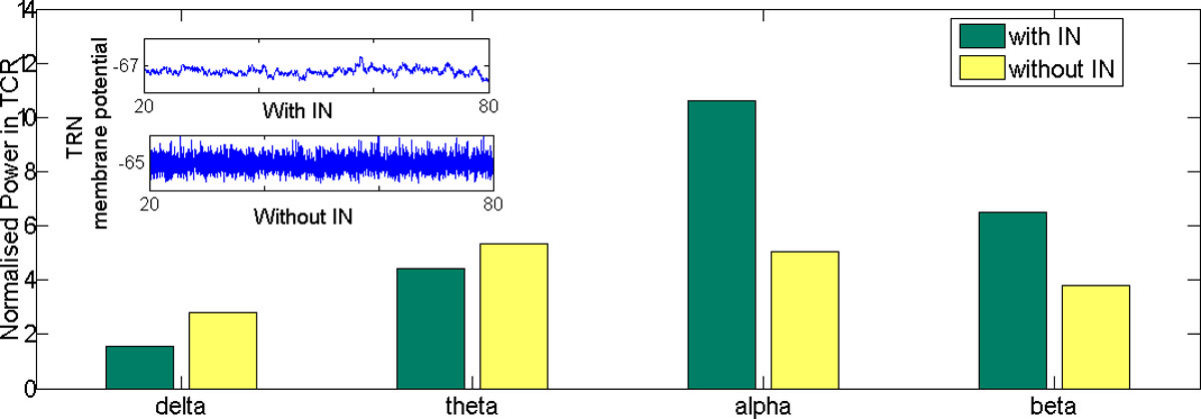# Studying the effects of thalamic interneurons in a thalamocortical neural mass model

**DOI:** 10.1186/1471-2202-15-S1-P219

**Published:** 2014-07-21

**Authors:** Thomas Bond, Simon Durrant, Louise O’Hare, Daniel Turner, Basabdatta Sen-Bhattacharya

**Affiliations:** 1 School of Engineering, University of Lincoln, Lincoln, Lincolnshire LN6 7TS, UK; 2 School of Psychology, University of Lincoln, Lincoln, Lincolnshire LN6 7TS, UK

## 

**Fig. 1:**
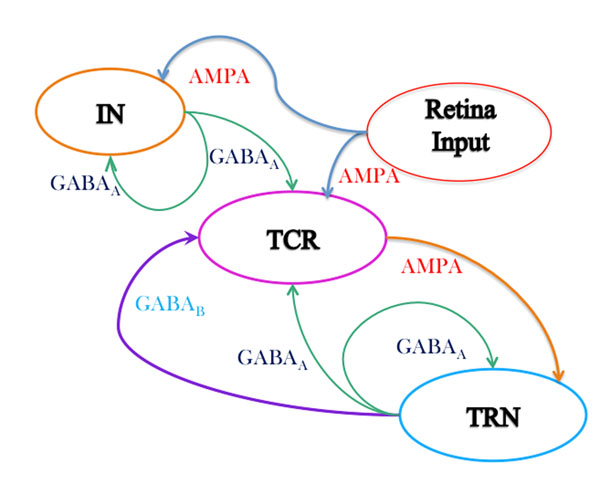


Neural mass models of the thalamocortical circuitry are often used to mimic brain activity during sleep and wakefulness as observed in scalp electroencephalogram (EEG) signals [1]. It is understood that alpha rhythms (8-13 Hz) dominate the EEG power-spectra in the resting-state [2] as well as the period immediately before sleep [3]. Literature review shows that the thalamic interneurons (IN) are often ignored in thalamocortical population models; the emphasis is on the connections between the thalamo cortical relay (TCR) and the thalamic reticular nucleus (TRN). In this work, we look into the effects of the IN cell population on the behaviour of an existing thalamocortical model containing the TCR and TRN cell populations [4]. A schematic of the extended model used in this work is shown in Fig.1. The model equations are solved in Matlab using the Runge-Kutta method of the 4^th^/5^th^ order. The model shows high sensitivity to the forward and reverse rates of reactions during synaptic transmission as well as on the membrane conductance of the cell populations. The input to the model is a white noise signal simulating conditions of resting state with eyes closed, a condition well known to be associated with dominant alpha band oscillations in EEG e.g. [5]. Thus, the model parameters are calibrated to obtain a set of basal parameter values when the model oscillates with a dominant frequency within the alpha band. The time series plots and the power spectra of the model output are compared with those when the IN cell population is disconnected from the circuit (by setting the inhibitory connectivity parameter from the IN to the TCR to zero). We observe (Fig. 2 inset) a significant difference in time series output of the TRN cell population with and without the IN cell population in the model; this in spite of the IN having no direct connectivity to and from the TRN cell population (Fig. 1). A comparison of the power spectra behaviour of the model output within the delta (1-3.5Hz), theta (3.75-7.5Hz), alpha (7.75-13.5Hz) and beta (13.75-30.5Hz) bands is shown in Fig. 2. Disconnecting the IN cell population shows a significant drop in the alpha band power and the dominant frequency of oscillation now lies within the theta band. An overall ‘slowing’ (left-side shift) of the power spectra is observed with an increase within the delta and theta bands and a decrease in the alpha and beta bands. Such a slowing of EEG is a signature of slow wave sleep in healthy individuals, and this suggests that the IN cell population may be centrally involved in the phase transition to slow wave sleep [6]. It is also characteristic of the waking EEG in Alzheimer’s disease, and may help us to understand the role of the IN cell population in modulating TCR and TRN cell behaviour in pathological brain conditions.

**Fig. 2:**